# Placental Amino Acids Transport in Intrauterine Growth Restriction

**DOI:** 10.1155/2012/972562

**Published:** 2012-07-11

**Authors:** Laura Avagliano, Chiara Garò, Anna Maria Marconi

**Affiliations:** Department of Obstetrics and Gynecology, DMSD San Paolo Hospital Medical School, University of Milano, 20142 Milano, Italy

## Abstract

The placenta represents a key organ for fetal growth as it acts as an interface between mother and fetus, regulating the fetal-maternal exchange of nutrients, gases, and waste products. During pregnancy, amino acids represent one of the major nutrients for fetal life, and both maternal and fetal concentrations are significantly different in pregnancies with intrauterine growth restriction when compared to uncomplicated pregnancies. The transport of amino acids across the placenta is a complex process that includes the influx of neutral, anionic, and cationic amino acids across the microvilluos plasma membrane of the syncytiotrophoblast, the passage through the cytoplasm of the trophoblasts, and the transfer outside the trophoblasts across the basal membrane into the fetal circulation. In this paper, we review the transport mechanisms of amino acids across the placenta in normal pregnancies and in pregnancies complicated by intrauterine growth restriction.

## 1. Introduction

The placenta represents a key organ for fetal growth as it acts as an interface between mother and fetus regulating the fetal-maternal exchange of nutrients, gases, water, ions, and waste products; moreover, it is capable of metabolic, immunologic, and endocrine functions. 

In humans, the hemochorial placenta includes the syncytiotrophoblast (a continuous, uninterrupted, multinucleated surface that covers the villous tree), the cytotrophoblast (a second layer of mononucleated trophoblasts that become discontinuous as pregnancy progresses), the connective tissue of the villous tree, and the endothelium of the fetal capillaries. During pregnancy, the placenta grows in volume, weight, and in terms of development and maturation of the type of villi, to allow the optimal fetal-maternal exchange [1]. Terminal villi are the final ramifications of the villous tree, characterized by a very thin syncytiotrophoblastic layer facing the fetal capillaries with the least maternal-fetal distance. The syncytiotrophoblast, therefore, is the key structure in regulating transplacental exchange across its maternal and fetal facing: the microvillous plasma membrane (MVM) and the basal membrane, respectively (BM).

During pregnancy, amino acids represent one of the major nutrients for fetal life; they are important precursors for fetal development and growth, for the biosynthesis of proteins, nucleotides (purine and pyrimidine), neurotransmitters, and so forth. The transport of amino acids across the placenta is a complex process mediated by transporters located on the MVM and BM of the syncytiotrophoblast. 

The purpose of this paper is to review the transport mechanisms of amino acids across the placenta in normal pregnancies and in pregnancies complicated by intrauterine growth restriction (IUGR).

## 2. Maternal and Fetal Concentrations

The fetal plasma concentration of most amino acids does not change during pregnancy and is significantly higher than maternal concentration [2–5], indicating an active transport across the placenta, from the maternal to the fetal circulation. In addition, in normal pregnancies, between maternal and fetal concentrations, there is a significant linear relationship for most amino acids leading to an increase in the umbilical venous concentration as maternal concentration increases [5, 6].

## 3. Placental Amino Acid Transport and**** Metabolism

The concentration of free amino acids in the placental tissue is higher than the concentration both in fetal and maternal plasma [2]; the placenta not only transports amino acids to the fetus, but its production and/or utilization of an amino acid plays an active role in determining its flux into the fetal circulation. *In vivo* animal studies have shown that the placenta is an extremely active organ metabolically with a very high rate of protein turnover and with some amino acids produced/utilized at very high rate. In addition, the presence of interorgan cycles of some nonessential amino acids between the placenta and fetal liver has been demonstrated: fetal glutamine and glycine are metabolized in the fetal liver and released to the placenta as glutamate and serine, respectively [7–11]. Studies performed in pregnant women with stable isotopes suggest that a similar interaction is present also in human pregnancies [12].

Many types of amino acid transport systems have been identified in the placenta [13] (Table 1). Each transporter is highly stereospecific, but different transporters have overlapping substrate specificity, with the possible compensation of one transporter activity by another [14].

**Figure 1:**
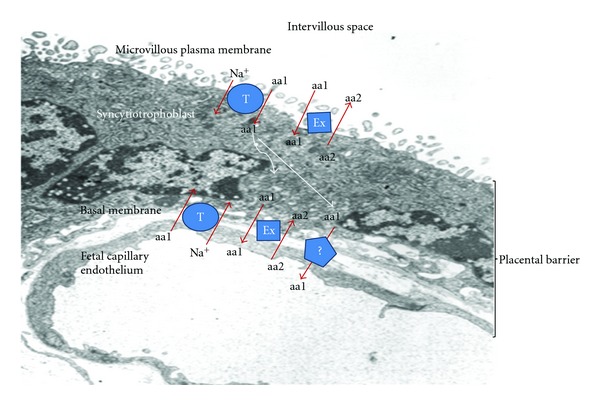
Mechanisms of amino acid transport. Na^+^-dependent transporters (T) permit the uptake of amino acids (aa) into the cell; amino acids are actively transported against a concentration gradient, using a Na^+^ gradient maintained by the Na^+^/K^+^ ATPasi. Amino acid exchangers (Ex) mediate the passage of amino acids by switching the position of one amino acid (aa1) from outside the cell and with one other (aa2) inside the cell. The transport across the basal membrane is poorly understood: may be mediate by amino acid exchangers (swapping one amino acid within the syncytiotrophoblast for one in the fetal capillary) or a nonexchanger passage may exist (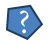) such as facilitate diffusion. (electron microscopy image: courtesy of GP Bulfamante; Diagram of amino acids transport modified from [15]).

Two major classes of amino acids transporters have been described: Na^+^-dependent transport systems (that mediate amino acids influx and lead to increased concentration of amino acids within the cell) and Na^+^-independent systems [19–21] (Table 1, Figure 1).

As mentioned earlier, the syncytiotrophoblast is the key structure in regulating transplacental amino acids passage. The transport through the syncytiotrophoblast includes the influx of neutral, anionic, and cationic amino acids across the MVM, the passage through the cytoplasm of the trophoblasts, and the transfer outside the trophoblasts across the basal membrane into the fetal circulation. Placental amino acids transporters are present both at the microvillous and basal membrane levels. Whereas the transport across the MVM has been well studied, that across the BM is less understood: the transport into the MVM of the syncytiotrophoblast almost always requires energy to act against the concentration gradient (Na^+^-dependent transport systems); on the contrary, in the outflux of amino acid across the BM, Na^+^-independent systems have an important role [21]. The transport across the BM may be mediated by amino acid exchangers (that take one amino acid molecule from outside the cell and one from inside the cell and switch their position); moreover, recently, the presence and efficacy of some efflux transporters (TAT1, LAT3, LAT4) in the human BM have been reported in isolated perfused human placental cotyledons [22] suggesting that facilitate diffusion is possible across the syncytiotrophoblast basal membrane.

Furthermore, during pregnancy, an adaptive response to different fetal nutrient demands seems possible [23], based on the evidence of changes in placental transporters expression and activity during the course of gestation: it has been shown that the activity of system A increases [24]. In addition, it has been shown that, during pregnancy, the same amino acid may be transported through different systems, contingent to which membrane is being crossed: in term placentas, L-arginine transport across the microvillous membrane preparations seems to occur through both the y^+^ and y^+^L systems, while, in the basal membrane, transport may be restricted to the y^+^L system [25]. Altogether, these observations point to the complex interactions between the developing microvillous and basal membrane within the trophoblast and between the maternal and fetal circulations, to facilitate an increase in nutrient delivery to warrant the demand of the growing fetus [26]. In other words, the placenta acts as a “nutrient sensor” regulating its transporter function [18].

## 4. Intrauterine Growth Restriction

Intrauterine fetal growth is determined by a balance between fetal genetically determined growth potential and maternal-placental nutrients supply [27]. Some factors influence fetal nutrition: maternal nutrition and metabolism, utero-placental blood flow, placental size, and placental transfer capacity [28]. In pregnancies complicated by intrauterine growth restriction (IUGR), all these factors can be affected [29].

### 4.1. Maternal and Fetal Concentrations

The concentration of most amino acids is significantly decreased both in the umbilical artery and vein of IUGR pregnancies when compared to normally grown babies [5, 6, 30, 31]: in particular, small for gestational age fetuses have significantly lower concentrations of the essential branched chain amino acids valine, leucine, and isoleucine [5]. Furthermore, in IUGR, the maternal concentration of most essential amino acids is significantly higher than in pregnancies with appropriate for gestational age (AGA) fetuses, likely as a result of a maladaptation to pregnancy with a deficient hormone production: this observation, together with the presence of lower fetal amino acid concentrations in intrauterine growth restriction, leads to significantly lower fetal-maternal differences in these pregnancies [6, 30]. 

Moreover, in IUGR pregnancies, increasing the maternal concentration of amino acids leads to an increased umbilical uptake of some of the amino acids to the fetus but with no evidence of a change in the uptake of the essential amino acids valine, phenylalanine, lysine, histidine, and threonine suggesting the presence of competition for the same transporter across the placenta that might block transport [32]. 

Recently, we have also shown that the maternal concentration of most amino acids is significantly increased within 48 hours after the administration of antenatal corticosteroids, and this determines that the concentrations of phenylalanine, methionine, threonine, valine, leucine, serine, glycine, alanine, glutamine, and proline are also significantly increased both in the umbilical vein and artery when compared to controls. However, the umbilical venoarterial difference of total amino nitrogen was not significantly different from zero: overall, the results of this study suggest that, in IUGR pregnancies, corticosteroids not only increase maternal protein catabolism but increase fetal protein catabolism as well. In addition, despite an increase in protein catabolism, those amino acids with relatively large bidirectional flux across the placenta, such as leucine and phenylalanine, do not exhibit large increases in fetal concentration; on the contrary, other amino acids, with very little bidirectional flux, such as alanine and threonine, are trapped within the fetal circulation leading to the large increase in their concentrations [33]. Whether corticosteroids have a direct effect on the human placental amino acid transport systems, as it has been shown in the mouse placenta [34], needs to be determined.

### 4.2. Placental Amino Acid Transport and Metabolism

Studies we have performed in human pregnancies at the time of fetal blood sampling, during a constant infusion of L-[1-13C]-leucine, have also shown that the fetomaternal leucine enrichment ratio progressively decreases in IUGR based on clinical severity [35]: this suggests not only that the transplacental flux of leucine is impaired but also a possible increased protein catabolism in these pregnancies [35]. In addition, if injected as a bolus into the maternal circulation of IUGR pregnancies, the fetomaternal enrichment ratio of two essential amino acids, leucine and phenylalanine, is significantly lower than in AGA pregnancies, again suggesting an impaired placental flux, whereas no differences are present for the nonessential amino acids, glycine, and proline [36].

However, as recently reviewed [21], some external factors may regulate the activity of amino acid transporters such as oxygen level [37], reactive oxygen species [38], insulin [37], leptin [39], and angiotensin II [40]. Therefore, it remains to be established whether the impairment of the amino acid transport system is the cause or the consequence of IUGR: we have shown that placental MVM system A activity not only is lower in IUGR compared with normal pregnancies but is also related to the severity of IUGR [41].

*In vivo* studies of placental amino acid transport and metabolism in the ovine heat-stress model of IUGR have shown a reduced flux of maternal leucine into the placenta and fetus [42]: this reduction is due to the reduction in placental and fetal mass and is accompanied by a decreased uteroplacental utilization of leucine. In addition, since uteroplacental oxygen and glucose consumption rates per gram of tissue remain within normal limits, the decrease in leucine utilization is not due to the general decline in metabolic rate [42]. In the same model, decreased fetoplacental threonine flux into the fetus and decreased fetoplacental threonine oxidation rate have been demonstrated indicating a downregulation of placental amino acids transport [43].

In severe IUGR fetal lambs (placental and fetal weights reduced by 40–60%), it has been shown that umbilical oxygen, glucose, and essential amino acid uptakes are significantly reduced compared to control animals whereas there are no differences in moderate IUGR (placental and fetal weights reduced by 25%) [44]. Two possible explanations have been proposed for these difference: first, since the placental diffusional exchange capacity of the severe IUGR fetus is significantly reduced, compared to AGA and moderate IUGR, changes in placental permeability and surface area might act as an impediment to control value uptakes per unit fetal weight; second, an upregulation of specific placental transport systems might be present since the mRNA expression of system L light chain components, LAT-1 and LAT-2, in severe IUGR is not different from control placentas, whereas it is significantly elevated in moderate IUGR [44]. 

*In vitro *studies of the human transport of amino acids have been performed [26]: in vesicle obtained from IUGR placentas, a reduced uptake of leucine and lysine has been reported, indicating a reduction of number or activity of the neutral and cationic amino acids transporters [45]; a decreased transport of taurine in isolated MVM has also been observed [46], suggesting a reduced activity of *β* amino acid transporters. Furthermore, in MVM and BM vesicles from IUGR placentas, a decreased activity of system A (a sodium-dependent neutral amino acid transporter) has been shown [47–49], and the decreased activity of system A in MVM has been also related to the severity of IUGR [41].  Table 2 summarizes the alteration of amino acid transporters in the human placenta in IUGR pregnancies.

## 5. Fetal Programming

Evidence suggests that intrauterine fetal life is the mirror of what happens to human health in adult life [50]: abnormal intrauterine fetal growth (in excess or defect) is associated with the development of metabolic syndrome in adult life [50]. 

Epigenetic dysregulation may be the link between intrauterine events and adult disease; data from animal models suggest that nutrition in pregnancy could result in epigenetic modification [51]: a low-protein diet during pregnancy activates the placental amino acid response pathway in rats and programs the growth capacity of offspring [52]; moreover, in mice, maternal undernutrition alters the placental phenotype by adapting the expression of glucose and amino acids transporters to support fetal growth [53].

The metabolism of the fetus is adaptive and programmed to respond as expected to postnatal life [54]. Furthermore, as mentioned earlier, the placenta is a nutrient sensor [18]: if it senses an environment with low nutrient levels (deficit of maternal supply, such as in maternal undernutrition, alteration in substrate and oxygen level in maternal blood, alteration of placental blood flow), it increases its transport activity to allow normal fetal growth, by increasing the passage of nutrients from the maternal to fetal circulation; on the other hand, if there is an insufficient nutrient supply at the maternal side, the placenta may decrease its transport capacity, adapting fetal growth to a lower level, in order to reduce fetal (and postnatal) demand [55]. In addition, the placenta may modulate its transport activity even when it perceives an environment with a high nutritional content, as in gestational diabetic pregnancies. In these cases, an upregulation of glucose and amino acids transporters has been observed [55]. 

If the intrauterine environment may influence the epigenetic regulation, it is theoretically conceivable that impaired placental transport function could affect epigenetic regulation. In other word, the placenta may adapt fetal metabolism, and, therefore, the transport function of the placenta could be considered a “programming agent.”

## Figures and Tables

**Table 1:** Amino acids transport systems in the human placenta.

Transport system	Protein	Localization	Substrate
Na^+^-dependent systems			

A	SNAT1, 2, 4	MVM, BM	Neutral amino acids
ASC	ASCT1, 2	BM	Neutral amino acids
*β*	TAUT	MVM, BM	Taurine
N	SN1	MVM (*contested in humans*)	Histidine, asparagine, glutamine
X_AG_^−^	EAAT1–4	MVM, BM	Anionic amino acids
GLY	GLYT1	MV	Glycine and sarcosine
B^0,+^	ATB^0,+^	?	Cationic and neutral amino acids

Na^+^-independent systems			

L	LAT1, 2, 4/4F2hc	MVM, BV	Neutral amino acids, branched-chain amino acids, and tryptophan
y^+^	CAT1, 4	MVM, BV	Cationic amino acids
y^+^L	y^+^LAT1/4F2hc	MVM, BM	Cationic amino acids (neutral amino acids in the presence of sodium)
b,^0,+^	rBAT	BM	Cationic and neutral amino acids
T	TAT1	BM	Aromatic amino acids
asc	asc1/4F2hc	BM?	Small neutral amino acids and D-serine

MVM: microvillous membrane.

BM: basal membrane.

Modified from [15–17].

**Table 2:** Alteration of amino acids transporters in the IUGR human placenta.

Transport system	MVM	BM
Taurine	−	=
Lysine	=	−
Leucine	−	−
System A	−	=

−: decreased; =: unaltered transporter activity; MVM: microvillous membrane; BM: basal membrane (modified from [18]).
